# The Relationship Between TikTok Usage and Executive Function Is Mediated by Problematic Social Media Use

**DOI:** 10.3390/bs15121748

**Published:** 2025-12-18

**Authors:** Jessica Golding, Anya Rallison, Kyra Zhang, Aisha Awan, Francisco Romero, Jillia Lacbain, Samuel Lee, Sahar Momand, Lilian Azer, Weiwei Zhang

**Affiliations:** 1 Department of Psychology, University of California, Riverside, 900 University Ave, Riverside, CA 92521, USA; 2 Department of Neurobiology and Behavior, University of California, Irvine, 260 Aldrich Hall, Irvine, CA 92697, USA

**Keywords:** working memory, problematic social media use, TikTok, BRIEF scale, executive function

## Abstract

Social media is deeply integrated into life, offering new opportunities for learning and communication. However, excessive use has been linked to negative effects on well-being and cognitive functions. A rapidly growing platform, TikTok has been a focal point of controversy due to concerns over its short-form content. The present study investigates the relationship between TikTok usage, problematic social media use (PSMU), and executive function (EF) to distinguish platform-specific features from broader patterns of problematic use. A survey conducted with 346 college students measured EF, TikTok use intensity, PSMU, and mental health variables. The findings indicate that while PSMU and TikTok use correlate with EF dysfunction, TikTok use is not a significant predictor when PSMU and mental health factors are statistically accounted for. Mediation analysis suggests that PSMU mediates the relationship between TikTok use and EF impairment, indicating that problematic media use—rather than platform-specific characteristics—relates to poor cognition connected to social media. These results contribute to the growing literature on digital media’s cognitive effects, underscoring the importance of addressing problematic usage patterns rather than focusing on individual platforms. Additionally, the association of PSMU with EF impairments and poor mental health reinforces the need for interventions targeting excessive social media engagement.

## 1. Introduction

Social media has evolved information transfer, enabling the sharing of content globally. Among the slew of platforms, TikTok emerged as highly influential, leveraging sophisticated algorithms to grab users’ attention with brief, engaging short-form video content (SFV). Initiating a nationwide debate, media outlets report that some fear that user data is accessed by foreign entities (McMahon, 2024), while others are reporting advocacy for limiting young audiences’ access (Butler & Taylor, 2024). The scrutiny revolves around mental health and developmental challenges in younger generations who are exposed to excessive consumption. TikTok has become the spearhead for the worry, relying heavily on user-curated, continuous streams of recommended SFVs. However, rather than placing blame on TikTok, careful consideration of problematic usage should be taken. Working memory (WM), attention, and inhibition have been directly, and indirectly through mental health, impacted by problematic social media usage (PSMU) (Reed, 2023; Almarzouki et al., 2022). Understanding how social media relates to poor cognitive outcomes, such as low EF, can help guide preventative measures and a deeper understanding of new technologies’ influence on the mind. The current work examines TikTok usage against PSMU to explore the underlying factor in social media’s relationship to poor executive function (EF). With this, we can gain insight into where this relationship stems from to combat decreased cognition and work to isolate beneficial features of social platforms.

Supporting EF’s role in goal-directed behavior are inhibition, WM, and cognitive flexibility (Diamond, 2012). EF marks cognitive health as it is largely correlated to overall life success (for a detailed review on EF, see Diamond, 2012). WM—specifically updating and monitoring—attention, and emotional control coalesce into EF (Roth et al., 2005; Best & Miller, 2010), and are all are strained by overused social media. Concern for TikTok’s effect on cognition is specifically focused on its SFV, as it may pose a greater risk compared to traditional platforms.

TikTok sets itself apart from past media platforms by using highly curated SFV. SFV is linked to triggering distraction, memory attrition (Asif & Kazi, 2022), boredom (Tam & Inzlicht, 2024), WM dysfunction (Sha & Dong, 2021), and overall cognition (Nguyen et al., 2025). Additionally, SFV may lead to decreased inhibition through its previously documented connection to the orbital frontal cortex’s increased usage (Gao et al., 2025). TikTok also provides SFV through personalized algorithms, which have been seen to activate default mode networks and the ventral tegmental area, linking to attentional issues (Su et al., 2021). Both regions contribute to EF (Nejati et al., 2018), explaining the claims driving the debate about TikTok’s connection to poor EF. However, with impacted impulse control, default mode network, and risky behavior, TikTok’s connection to EF decline is perhaps better explained through PSMU of its content. SFV and algorithms pose concern for how TikTok cultivates PSMU in young adults. Past work confirms suspicions that TikTok is seemingly addictive due to the quality of content and general app experience (Qin et al., 2022; Caponnetto et al., 2025), leading to higher PSMU.

Patterns of impulsive, excessive, disruptive, and compulsive usage indicate PSMU (Sun & Zhang, 2021). A total of 30–40% of adolescents claim symptoms of PSMU (Shahid et al., 2024), making them vulnerable to poor mental health (Cunningham et al., 2021; O’Day & Heimberg, 2021; Shahid et al., 2024), fractured relationships, and thwarted aspirations (Sun & Zhang, 2021). Sleep quality, a known predictor of EF (Xie et al., 2019), is directly decreased by problematic phone usage (Cui et al., 2021). Overactivation of the anterior cingulate cortex in those with internet addiction causes concern for cognitive monitoring and control (Dong et al., 2012). WM, a temporary and limited capacity memory (W. Zhang & Luck, 2008), has also been previously seen to be disrupted by PSMU through post-offloading (Tamir et al., 2018), memory failures, and increased negative affect (Sharifian & Zahodne, 2021; Frein et al., 2013). PSMU also creates heightened mind wandering (Lian et al., 2022), further contributing to WM failure as drifted attention damages filtering and prioritizing (Rummel & Boywitt, 2014; Hollis & Was, 2016; Awh et al., 2006). The EF skills of language comprehension, planning, and decision making are all supported by WM (Cowan et al., 2005), demonstrated broadly by WM predicting compliance to COVID-19 social distancing (Azer et al., 2024; Xie et al., 2020; W. Zhang et al., 2020). Additionally, the EF components of impulsivity (Reed, 2023), inhibition (Nie et al., 2016; Murphy & Creux, 2021), filtering (K. Zhang et al., 2023), and attention span (Husain et al., 2024) are all exacerbated by PSMU, suggesting that EF is at risk.

Moreover, PSMU heightens risks for mental health, including emotional instability (Twenge & Campbel, 2018), anxiousness (S. Li et al., 2025), and depression (Cunningham et al., 2021). The influx of seeing unattainable lifestyles and unrealistic expectations leads to low self-esteem and worsened depression (Samra et al., 2022). Longitudinal work captures exacerbated mental health issues in connection to PSMU specifically, rather than typical interaction with social media (J. B. Li et al., 2018). PSMU’s parallels to addiction create feelings of perceived helplessness and hyperfixation on negative stimuli connected to depression (Levens & Gotlib, 2010; J. Zhao et al., 2023), along with its other symptoms (Rose & Ebmeier, 2006; Xie et al., 2018, 2019, 2020), and symptoms of anxiety (Darke, 1988; Sternberg et al., 2018; Xie et al., 2020), which work to significantly reduce WM and EF. With our prediction that PSMU should be the driver of social media’s connection to poor EF, we seek to further understand how PSMU might influence EF. Therefore, understanding PSMU’s connection to EF through mental health can help pinpoint multifaceted complexities of their relationship.

The current work seeks to uncover whether PSMU harbors the explanatory power for social media’s connection to poor cognition, or if TikTok’s connection to lowered EF is fostered by the platform rather than behavior. If links to poor EF are driven by platform, TikTok should independently lower EF when controlling for PSMU and mental health factors. However, if PSMU better explains the variance, then TikTok use should become insignificant. Due to TikTok content’s connection to problematic use risk factors and problematic behavior risk factors connected to EF, WM, and mental health, it is predicted that the latter option is prevalent, causing the connection TikTok has to EF to be mediated through PSMU. Additionally, due to mental health issues being exacerbated by PSMU and symptomology being problematic to EF, PSMU’s relationship to EF will be mediated through depression and anxiety.

## 2. Materials and Methods

### 2.1. Transparency and Openness

We fully disclose how our sample was obtained, and how data were excluded from the final data used. All measures are reported, and any manipulations to the raw data are explained. The analysis code, along with the data and research material, is available for use upon request.

### 2.2. Participants

Participants were recruited from the University of California, Riverside (UCR), using a SONA student pool. SONA is an online study recruitment platform, where students receive course credit for participating in studies. Initially, there were 357 participants; however, due to missing data, some were excluded, leaving 346. After excluding cases, 57% of participants were female, with ages ranging from 17 to 51 years old, a mean of 19.90, and a standard deviation of 2.66. The sample demographics of participants are shown in Table 1.

### 2.3. Measures

#### 2.3.1. BRIEF-A-SI

The Behavior Rating Inventory Executive Function-Adult self-interpretive version (BRIEF-A; Roth et al., 2005) measured EF in college students. The scale consists of 75 three-point Likert scale questions of “never”, “sometimes”, and “often” regarding 9 different subsections of EF. These 9 categories are inhibition, shifting, emotional control, self-monitoring, initiating, WM, planning, task monitoring, and organizational abilities. Questions asked participants to self-report EF, with questions like “I need to be reminded to begin a task even when I am willing”. As a measure of EF, we acknowledge that capturing failures of cognition in self-report forms is challenging. However, due to the high internal consistency of the full scale (Cronbach alpha = 0.96) and previously documented accuracy in capturing EF (Erkkilä et al., 2018; Xie et al., 2020; Azer et al., 2024), we feel comfortable addressing scores as EF (Roth et al., 2013).

#### 2.3.2. SMAS-SF

The Social Media Addiction Scale-Student Form (SMAS; Sahin, 2018) captured PSMU. While this scale is intended to measure addiction, controversy surrounding social media addiction as a true addiction is ever-present (Hall, 2024; Tsilosani et al., 2023). Therefore, this survey is used to capture PMSU, rather than diagnose those who score higher with an addiction disorder. Questions revolve around unhealthy social media use, asking if “a life without social media becomes meaningless for me” on a five-point Likert scale. The 29 questions’ scores range from “strongly disagree” to “strongly agree”. Items can be further divided into four categories of tolerance, communication, problems, and information, with high internal consistency in the data (Cronbach’s alpha = 0.91).

#### 2.3.3. TikTok Intensity

Our TikTok Intensity Scale (TTI) was adapted from the Multidimensional Facebook Intensity Scale (MFIS; Gabor et al., 2015) to measure overall TikTok use. Items capture intensity of usage, such that those with higher scores interact with TikTok as their main platform. Therefore, those with in-depth, frequent exposure to its platform should interact with platform features more than compared to casual users. Thirteen questions from the MFIS were changed to indicate that the measure was focused on TikTok rather than Facebook. For instance, the question “When I’m bored, I often go to Facebook” was changed to “When I’m bored, I often go to TikTok”. Questions were only changed to read “TikTok” in place of “Facebook” to maintain scale integrity. Responses were on a five-point Likert scale ranging from “strongly disagree” to “strongly agree” with subcategories of persistence, boredom, overuse, and self-expression. High internal reliability was captured in the data (Cronbach’s alpha = 0.91).

#### 2.3.4. PHQ-9

The Patient Health Questionnaire 9 (PHQ-9; Lowe et al., 2004) measured depressive symptoms and severity. Nine questions asked participants to reflect on the prevalence of symptoms such as “feeling tired and having little energy” on a four-point scale. Prompting participants to reflect on how often they have been bothered by the symptoms, the scale includes answers of “not at all”, “several days”, “more than half the days”, and “nearly every day”. Questions had good internal reliability (Cronbach’s alpha = 0.88).

#### 2.3.5. GAD-7

Anxiety symptoms were captured with the General Anxiety Disorder 7-item scale (GAD-7; Spitzer et al., 2006). Seven questions with the same response options as the PHQ-9 asked participants to judge how often they felt symptoms like “trouble relaxing”. The scale showed a high level of internal reliability in the study (Cronbach’s alpha = 0.90).

#### 2.3.6. Ethical Considerations

Participants were informed that their information would be kept private, with no identifying information being taken (i.e., name or email address). Each participant gave consent to participate and was able to withdraw at any point.

### 2.4. Procedure

#### 2.4.1. Data Collection

Data was collected from May to July 2023. Protocol was approved by UC Riverside IRB (HS 12-097 Attention and Memory, 20 September 2022–19 September 2023). Upon signing up for the study, a Qualtrics survey was sent to complete all questionnaires in one sitting. Qualtrics collected data via these online surveys in remote locations; therefore, no contact with participants was made. Before beginning, all participants provided consent or were allowed to exit the study.

#### 2.4.2. Analysis

Composites were formed by the simple addition of necessary questions, separately for each scale. To make results more interpretable, all variables were scaled, excluding mediation model analysis. All variables were continuous. Spearman’s correlations were run on all variables, as the variables’ data distributions were non-normally distributed. However, residuals were normally distributed, and therefore univariate regressions were run, looking at EF as the outcome and PSMU and TTI as individual predictors. Multivariate regressions first included all demographics, mental health measures, and TTI on EF. A second multivariate regression included all previous measures and PSMU on EF. An R-squared change test was used to determine if PSMU contributed any significant amount of additional variance to the model and if TTI maintained significance. As stated, the assumption of normality in residuals was met after assumption tests were run on each regression model, justifying the implementation of a parametric test. Finally, three mediation analyses were run. An initial mediation held PSMU as the mediator in TTI’s effect on EF. Then, two additional mediations included depression or anxiety’s mediation of PSMU’s relationship to EF.

## 3. Results

### 3.1. Descriptive Results

Correlation analyses investigated the linear relationship between all continuous variables in the present study (See Table 2 for the Correlation Matrix of Variables in the present study). Spearman correlation was used due to failed normality tests for all measures (e.g., Shapiro–Wilk test, EF: *p* < 0.001, PSMU: *p* = 0.016; TTI scores: *p* < 0.001).

### 3.2. Regression Models

**Figure 1:**
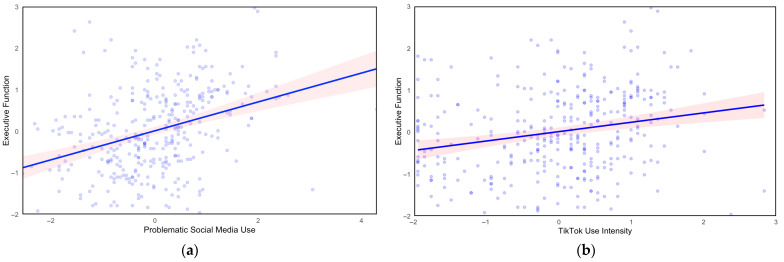
The solid line and the red shaded area indicate the linear fit of the data and its 95% confidence intervals, respectively. (**a**) Problematic social media use and executive function. PSMU significantly predicts poor EF. (**b**) TikTok use and executive function. TTI significantly predicts poor EF.

Two linear univariate regressions analyzed the relationship between the types of social media use independently of EF. First, PSMU significantly predicted EF (*b* = 0.47, *p* < 0.001), where, as the PSMU score increased, EF became worse (Table 3; Figure 1a). This relationship accounted for 11.7% of the variance in EF (*p* < 0.001). Second, TTI significantly predicted EF (*b* = 0.46, *p* < 0.001), where, with greater TikTok use intensity, EF functioning declined (Table 4; Figure 1b). This accounted for 4.6% of the variance in EF (*p* < 0.001).

A multivariate linear regression including all covariates and TTI on EF was significant (Table 5; Model 1) (*F*(5, 340) = 47.82, *p* < 0.001, R^2^ = 0.41), including significant variance explained by TTI (β = 0.12 [0.04, 0.21], *p* < 0.001), anxiety (β = 0.40 [0.11, 0.38], *p* < 0.001), and depression (β = 0.40 [0.26, 0.54], *p* < 0.001). The model that also included PSMU (Table 5; Model 2) was significant (*F*(6, 339) = 42.94, *p* < 0.001, R^2^ = 0.42); however, it showed that TTI no longer significantly predicted EF (β = 0.05 [−0.05, 0.14], *p* = 0.318) while controlling for PSMU and other covariates. Within the second model, PSMU (β = 0.16 [0.07, 0.26], *p* = 0.001) became significant, and anxiety (β = 0.26 [0.13, 0.40], *p* < 0.001) and depression (β = 0.35 [0.21, 0.49], *p* < 0.001) stayed significant predictors of EF. Additionally, an R-squared change test showed Model 2 significantly improved fit compared to Model 1, [ΔR^2^ = 0.019, *F*(1, 340) = 11.32, *p* < 0.001], suggesting that TTI failed to contribute additional unique variance in EF (Table 5). Therefore, controlling for PSMU and other covariates made TTI noninfluential on EF.

### 3.3. Mediation Models

**Figure 2:**
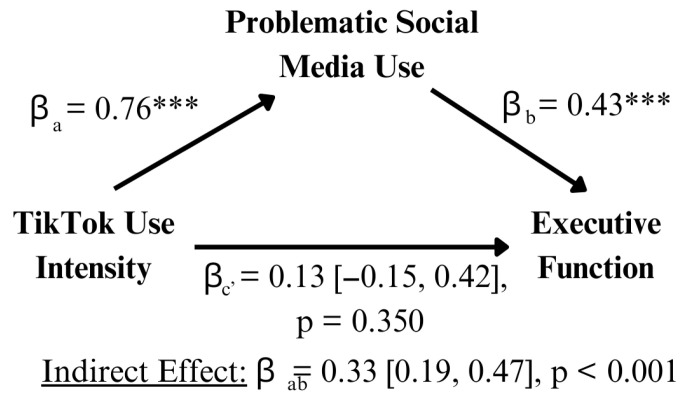
*** *p* < 0.001. Mediating effects of problematic social media use on TikTok’s relationship with executive function. The significant relationship of TikTok use with EF is fully mediated by PSMU. That is, TikTok users’ levels of EF are completely dependent on their level of problematic use, where higher levels lead to more dysfunction.

To explore TTI’s initial relationship to EF, a mediation analysis was conducted. PSMU was added as a potential mediator for TTI’s relation to EF (Figure 2). PSMU significantly mediated the relationship between TTI and EF (indirect effect: β = 0.33 [0.19, 0.47], *p* < 0.001). Furthermore, PSMU fully mediated the relationship between TTI and EF (β = 0.13 [−0.15, 0.42], *p* = 0.350). That is, TTI had no significant, direct effect on EF after the mediating effects of PSMU. Thus, the relationship between TTI and EF is understood and explained through PSMU behaviors.

**Figure 3:**
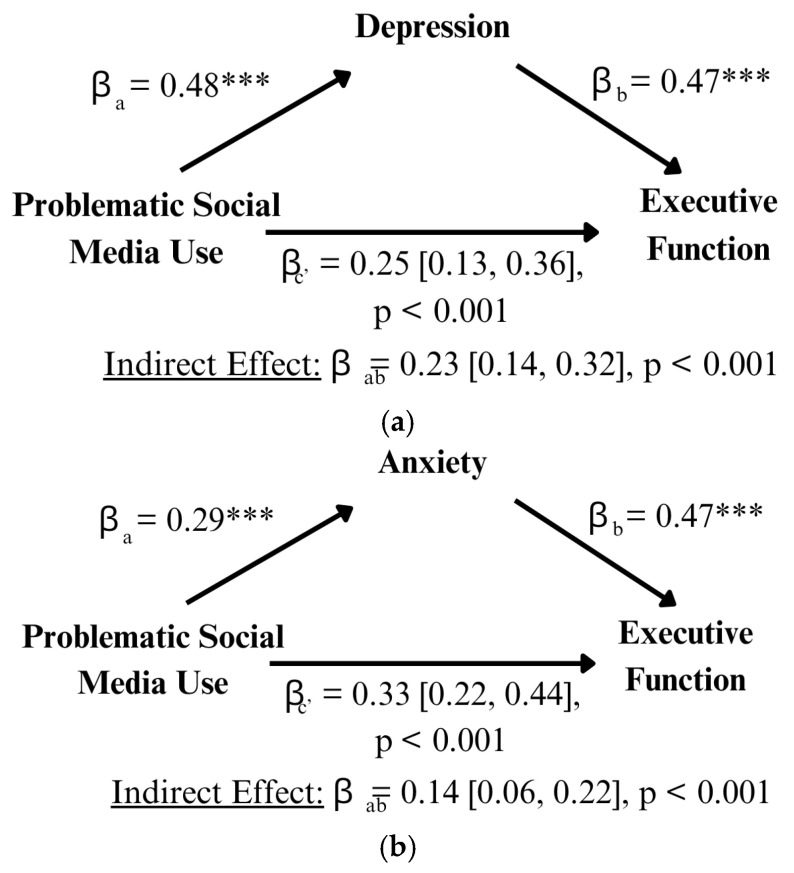
*** *p* < 0.001. (**a**) Mediating effects of depression symptoms on problematic social media use’s relationship with executive function. The significant relationship of PSMU with EF is partially mediated by depression symptoms. That is, problematic users’ levels of EF are partially dependent on the severity of their depression symptoms, where higher levels lead to poor EF. (**b**) Mediating effects of anxiety symptoms on the problematic social media use relationship with executive function: The significant relationship of PSMU with EF is partially mediated by anxiety symptoms. That is, problematic users’ levels of EF are partially dependent on the severity of their anxiety symptoms, where higher levels lead to poor EF.

A mediation of, separately, depression and anxiety on EF was run to account for mental health decline from PSMU and how it could further negatively impact cognition. Depression (Figure 3a) significantly partially mediated PSMU on EF (indirect effect: β = 0.23 [0.14, 0.32], *p* < 0.001), with still significant direct effects (β = 0.25 [0.13, 0.36], *p* < 0.001). Similarly, anxiety (Figure 3b) continued the same pattern, with a partially mediating effect (indirect effect: β = 0.14 [0.06, 0.22], *p* < 0.001), and a significant direct effect (β = 0.33 [0.22, 0.44], *p* < 0.001).

## 4. Discussion

It is important to highlight that the current results are correlational, so while directionality is explored, there are no concrete statistical implications in directional relationships. The current work seeks to dissect social media’s connection to cognition through EF, through both the lenses of platform-centered (i.e., TikTok) and use-centered (i.e., PSMU) relationships. The results supported predictions that increased TikTok consumption was connected to poor EF. However, multivariate models revealed that PSMU captured the explanatory variance of TikTok use and additionally mediated TikTok’s initial significant model. To gain a more multifaceted understanding of PSMU’s relationship to EF, a partial mediation showed that mental health decline holds partial explanatory power in the PSMU and EF relationship.

Both PSMU and TikTok use, when independent, contribute explanatory variance to EF, consistent with the literature that found excessive social media and SFV content could negatively relate to cognition. However, model comparison gave further insight into unique explanatory variance. Model 2, with both PSMU and TikTok use, added significant variance compared to Model 1, with only TikTok use. Furthermore, TikTok use failed to reach significance in Model 2. This finding suggests that declining EF is more strongly connected to PSMU compared to media platforms. This conclusion was further justified by PSMU’s full mediation effect.

Overindulgence tied to PSMU can foster cognitive overload, with task switching, novelty seeking, obsessions, and unpredictable rewards (Xie et al., 2020). Without controlling for patterns of use, it is easy to simplify this relationship down to the platforms connecting to poor EF. By including PSMU and thus explaining the variance contributed by TikTok use in our model, the relationship is more deeply captured to better understand how social media is connected to cognition under different contexts of use. This might help explain observed disconnects in technology and cognitive decline (Miller et al., 2023; Fleming, 2025). Cognition’s and PSMU’s relationship also seems to be cyclical, as a higher PSMU predicts lower attention and inhibition, which feeds into problematic use behaviors (Wang et al., 2024). Moreso than typical use, PSMU creates a feedback loop of poor cognition and impulsivity, which leads to even further abuse of media sites.

Additionally, TikTok’s correlation to PSMU hints towards heightened susceptibility to the problematic behaviors of frequent users. Unique dimensions of social sites have been shown to promote varied disorders (Rozgonjuk et al., 2020, 2021), possibly explaining this correlation, as the platform-specific features of TikTok could cultivate PSMU. SFV, the primary content presented on TikTok, has a previously documented connection to poor cognitive outcomes (Gao et al., 2025; Y. Zhao et al., 2025). However, individuals prone to PSMU might also happen to abuse TikTok as well, giving a counter-explanation for this correlation. This directionality should be further dissected to build on how PSMU explains TikTok’s connection to poor EF. However, the broad conclusion is that PSMU is involved with cognitive links to social media usage, whether the platform promotes these patterns or not.

Depression and anxiety’s partial mediation of PSMU and EF works to build a layered understanding. Heightened exposure to shallow connections and unrealistic creators creates disconnect and discontent, linked to increased mental health challenges. Additionally, boredom encourages constant swiping, creating paradoxical cycles of boredom (Tam & Inzlicht, 2024). Compulsive, habitual actions of PSMU create a lack of autonomy, in which self-determination theory posits that stress and negative affect arise to create depressed, anxious states. Past connections suggest PSMU might decrease EF through WM (Sun & Zhang, 2021; K. Zhang et al., 2023), which has previously had decreased functionality connected to poor mental health. This sub-relationship of WM to PSMU and mental health should be focused on.

Future work should seek to find direct causal connections, as current findings are correlational. The possible inclusion of longitudinal data with younger generations could help support directional claims. Current work by Kasturiratna and Hartanto (2025) helps support current findings and points toward cyclical patterns associating PSMU with cognition. The current work examining PSMU in a longitudinal framework is sparse, opening promising avenues for future theoretical development. Within cross-sectional data, it is important to consider biases in self-reported measures. Additional task-based EF and WM measures could provide a nuanced understanding of niche subtypes of EF and WM and provide control for any confounders associated with social desirability biases from participants. However, BRIEF is a validated, consistent measure compared to objective cognition (Erkkilä et al., 2018; Xie et al., 2020; Azer et al., 2024); thus, we confidently feel scores relay accurate EF levels to provide insights into cognition. Additional validation of the TTI scale should be considered as well, to ensure that it fully captures the intensity of TikTok usage. Our findings show that PSMU seemingly fully mediates TikTok’s contribution to EF, suggesting that TikTok does not contribute to EF independent of PSMU. However, this conclusion is limited to the current measures and sample. It is still possible that different measures of TikTok use may be related to different aspects or measures of EF (e.g., task measures). PSMU’s connection to addiction should be further debated, as reported feelings of withdrawal and relapse from self-prescribed media addicts occur (S. Li et al., 2025). Poor cognition, habituation, and the exacerbation of symptoms from poor mental health reflect patterns seen in other addictions. With regard to the negative light on TikTok, it increases misunderstandings of how poor behavioral patterns foster poor mental health and cognitive outcomes. While caution against heavy TikTok use should be given, poor patterns of social media use on any site are risky for cognition. Discouraging features of problematic usage while promoting curiosity and learning through social media is crucial to protect against negative outcomes while still reaping platform benefits.

## Figures and Tables

**Table 1:** Frequency of demographics.

Sex *	Count	Percentage	Ethnicity **	Count	Percentage	Age	Count	Percentage
1	148	42.77	1	12	3.47	17	1	0.29
2	198	57.23	2	39	11.27	18	71	20.52
			3	124	35.84	19	108	31.21
			4	135	39.02	20	83	23.99
			5	35	10.12	21	44	12.72
			6	1	0.29	22	20	5.78
						23	10	2.89
						24	1	0.29
						25	2	0.58
						28	1	0.29
						29	2	0.58
						37	2	0.58
						51	1	0.29

* For sex, 1 = male and 2 = female. ** For ethnicity, each number has its respective ethnicity: Black (1), Asian—south (2), Asian—east and southeast (3), Hispanic (4), White (5), and Pacific Islander (6).

**Table 2:** Spearman’s correlation of all variables.

	1	2	3	4	5	6
1. Executive Function	–					
2. TikTok Intensity	0.21 ***	–				
3. Problematic Use	0.34 ***	0.49 ***	–			
4. Depression	0.61 ***	0.15 **	0.29 ***	–		
5. Anxiety	0.57 ***	0.12 *	0.19 ***	0.79 ***	–	
6. Age	−0.07	−0.12 *	−0.08	−0.03	0.05	–
7. Sex	0.13 *	0.28 ***	0.12 *	0.20 ***	0.19 ***	0.05

Note: n = 346. For each correlation, * *p* < 0.05, ** *p* < 0.01, *** *p* < 0.001. Pearson’s correlations were run for items correlating with sex, due to no rank order being available to run a Spearman correlation. Due to the non-normality of other variables, corrective transformations to the data were run, which provided the same results as well.

**Table 3:** Predicting problematic social media use on executive function with simple regression.

	b	[95% CI]	β	[95% CI]	*p*
Intercept	84.64	[74.42, 94.85]			<0.001
Problematic use	0.47	[0.33, 0.61]	0.34	[0.24, 0.44]	<0.001
R^2^ (R^2^ adjusted)				0.117 (0.114)	<0.001

Note. A simple linear regression analysis was run, with EF as the outcome.

**Table 4:** Predicting TikTok’s effect on executive function with simple regression.

	b	[95% CI]	β	[95% CI]	*p*
Intercept	103.02	[95.05, 111.00]			<0.001
TikTok Intensity	0.46	[0.24, 0.68]	0.21	[0.11, 0.32]	<0.001
R^2^ (R^2^ adjusted)				0.046 (0.043)	<0.001

Note. A simple linear regression analysis was run, with EF as the outcome.

**Table 5:** Predicting executive function with multiple regression.

		Model 1			Model 2	
	β	[95% CI]	*p*	β	[95% CI]	*p*
Sex	−0.03	[−0.08, 0.08]	0.821	−0.02	[−0.11, 0.06]	0.635
Age	−0.05	[−0.14, 0.03]	0.165	−0.05	[−0.13, 0.03]	0.206
Anxiety	0.25	[0.11, 0.38]	<0.001	0.26	[0.13, 0.40]	<0.001
Depression	0.40	[0.26, 0.54]	<0.001	0.35	[0.21, 0.49]	<0.001
TikTok Use	0.12	[0.04, 0.21]	<0.001	0.05	[−0.05, 0.14]	0.318
Problematic Use				0.16	[0.07, 0.26]	0.001
R^2^ (R^2^ adjusted)		0.413	<0.001		0.432 (0.422)	<0.001
Comparison	ΔR^2^ = 0.019, F_(1, 340)_ = 11.32, *p* < 0.001					

Note. A multiple linear regression analysis was run with EF as the outcome. Model 2 includes the addition of problematic use. An R-squared change test was run to see if the inclusion of problematic use added meaningful significance to the model. The change test was significant, revealing that PSMU added a significant amount of variance to the model, leaving TikTok use insignificant.

## Data Availability

The data used is available from the corresponding author upon request.

## Abbreviations

The following abbreviations are used in this manuscript:

SFV	Short-form video content
WM	Working memory
EF	Executive function
PSMU	Problematic social media use
TTI	TikTok use intensity
